# Salvage Procedure for a Failed Tibiotalar Arthrodesis: A Trans‐Achilles Approach for Ankle Fusion Using a Posterior Plate—Case Reports and Literature Review

**DOI:** 10.1155/cro/5599494

**Published:** 2026-03-06

**Authors:** Ana Carolina Presas-Presas, Laura Chouza-Montero, Emma Escudero-Martínez, Miguel Prado-Leira

**Affiliations:** ^1^ Department of Orthopaedic Surgery, Complejo Hospitalario Universitario de Pontevedra, Pontevedra, Spain

**Keywords:** ankle arthrodesis, ankle osteoarthritis nonunion, talar necrosis, trans-Achilles approach

## Abstract

Treatment of ankle osteoarthritis (OA) is controversial. The early age of onset and the lack of consensus about the moment and type of surgical intervention among experts have pointed this disease as a matter for debate. Nonunion is a rather common complication after a tibiotalar arthrodesis. Several approaches have been described in the literature for failed ankle replacement, but there are fewer salvage alternatives for a failed arthrodesis. In this article, we present two clinical cases. The first one involves a 57‐year‐old woman diagnosed with primary ankle OA who underwent ankle arthrodesis (AA), which subsequently resulted in nonunion. A rescue procedure was suggested, and a trans‐Achilles approach using a plate for fixation was proposed. Arthrodesis was successfully achieved, and the patient remains satisfied with the outcomes. In the second case, we present a 44‐year‐old man with talar avascular necrosis after a AA. Due to persistent pain, a new arthrodesis via posterior approach was performed allowing good exposure of the necrosis area. No complications were reported after 2 years of postoperative follow‐up. As a conclusion, in our experience, trans‐Achilles rearthrodesis with a posterior plate after ankle fusion failure could be a suitable salvage option in patients in whom the anterior approach may involve soft tissue compromise.

## 1. Introduction

Nowadays, ankle osteoarthritis (OA) is estimated to affect 1% of the adult population [1]. The main etiology is posttraumatic, representing 75%–80% of all the cases, in contrast to knee or hip OA, where primary OA predominates. This posttraumatic cause explains why this disease affects younger people, with the socioeconomic impact that it involves [2]. Surgical treatment of end‐stage OA has been a subject of discussion in recent years. The latest studies point out the absence of differences between total ankle replacement (TAR) and ankle arthrodesis (AA) [3, 4] and choose the latter as the gold standard in physically high‐demanding patients [2].

Nonunion is a relatively frequent complication after an ankle fusion. The union rate after a primary arthrodesis is estimated to be between 74% and 93%, being lower in patients affected by some peripheral neuropathy such as Charcot‐Marie‐Tooth disease and diabetic neuropathy [5]. Risk factors related to different methods of fixation have not been reported [6]. Despite its high rate, there is no consensus about salvage treatment options for a clinical nonunion when an intramedullary nail was used.

On the other hand, avascular necrosis of the talus is a rare entity related in 75% of the cases to trauma, particularly with talus neck fractures [7]. Additional factors that seem to be related are alcoholism and corticosteroid usage [8].

Treatment includes a conservative approach in earlier stages and surgical options such as tibiocalcaneal arthrodesis with or without graft, tibiotalar arthrodesis (Blair fusion technique), and arthroplasty in advanced cases. Rates of nonunion are variable, rising up to 88% in some series [9].

In this paper, we are introducing two clinical cases involving a salvage treatment option for a failed AA with an intramedullary nail.

## 2. Case Presentation: Patient I

This case involves a 57‐year‐old nonsmoking female with high blood pressure and no other relevant medical history who suffered from ankle and rearfoot nontraumatic weight‐bearing pain. Following an exhaustive examination, she was diagnosed with primary OA and, after years of conservative treatment, an AA was suggested. The operation was carried out in a different hospital in May 2019, using an intramedullary nail (Phoenix, Biomet). Surgical wound healing complications were reported during the postoperative (PO) period; thus, thorough wound care was necessary and anterolateral ankle skin atrophy developed.

One year and a half after the surgery, she came back to the hospital complaining about persistent pain. A lack of fusion was identified in the x‐ray so a CT was performed, corroborating the diagnosis of nonunion (Figure 1). At that moment, the clinical situation involved a physically demanding patient with a painful failed AA without signs of infection, so another surgical procedure was attempted to achieve arthrodesis; due to the anterolateral skin atrophy, the chosen posterior trans‐Achilles approach used a plate for fixation.

Figure 1X‐ray 20 months after the first surgery, (a) anteroposterior and (b) lateral views showing absence of fusion of the tibiotalar and medial malleolus. CT (c) coronal and (d) sagittal images 20 months after surgery: subtalar OA is spotted.

**Figure:**
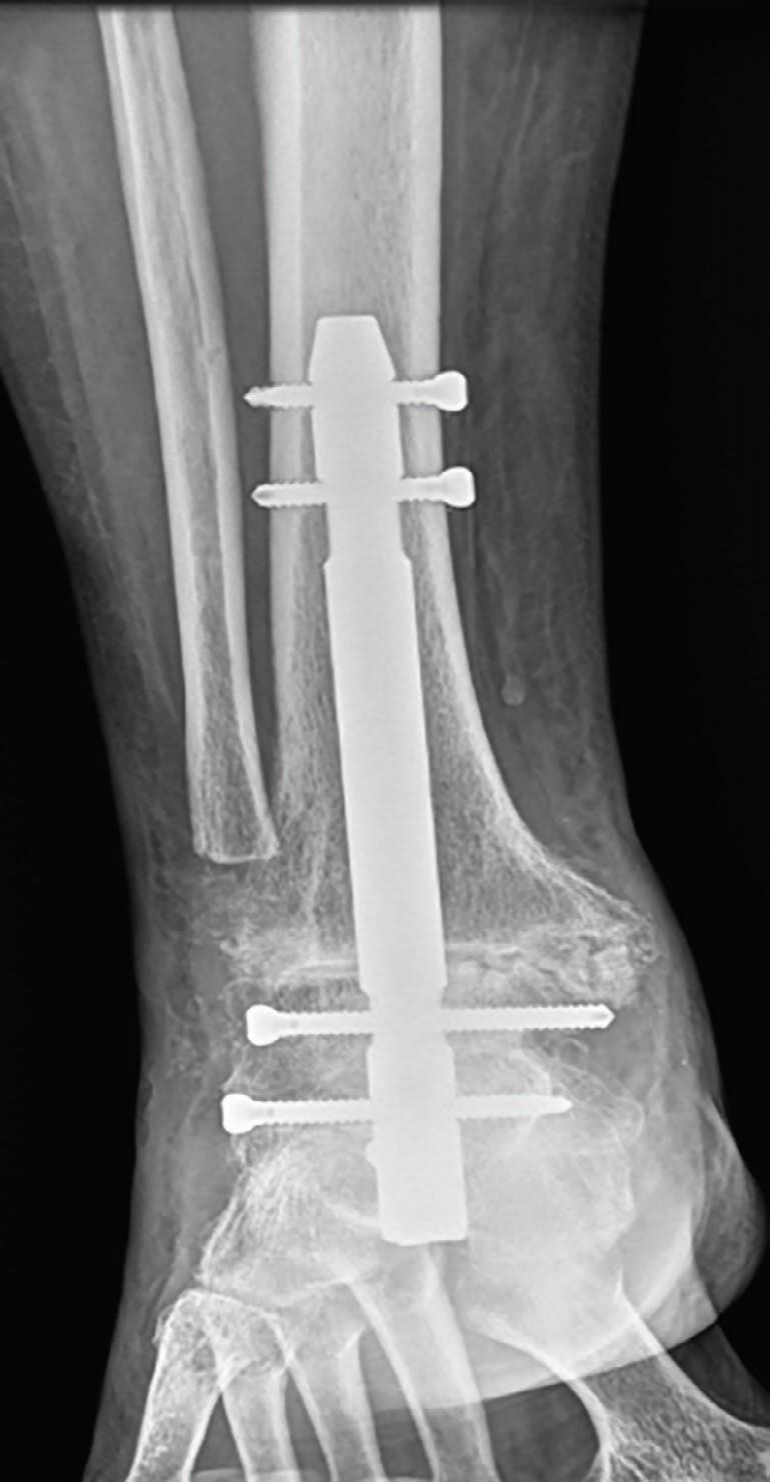
(a)

**Figure:**
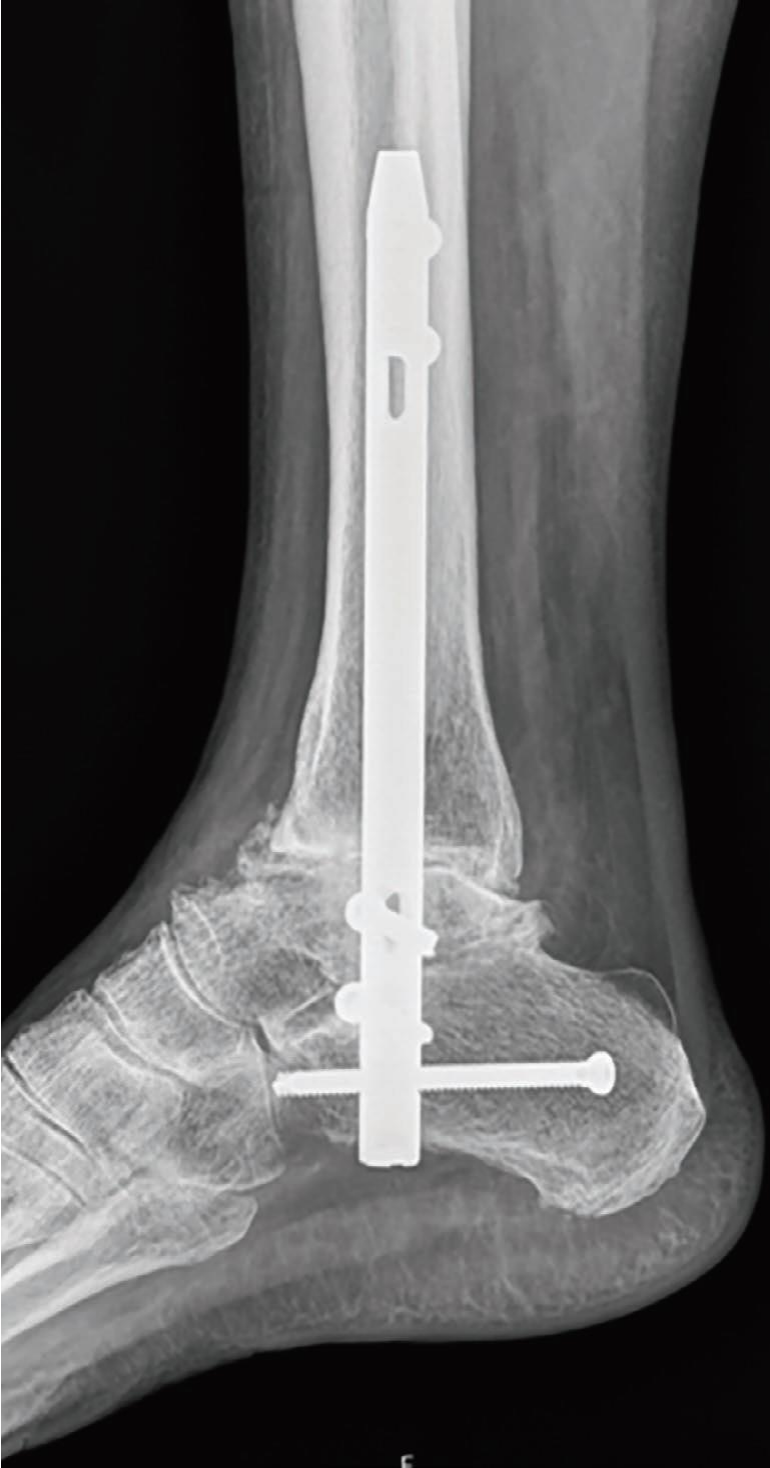
(b)

**Figure:**
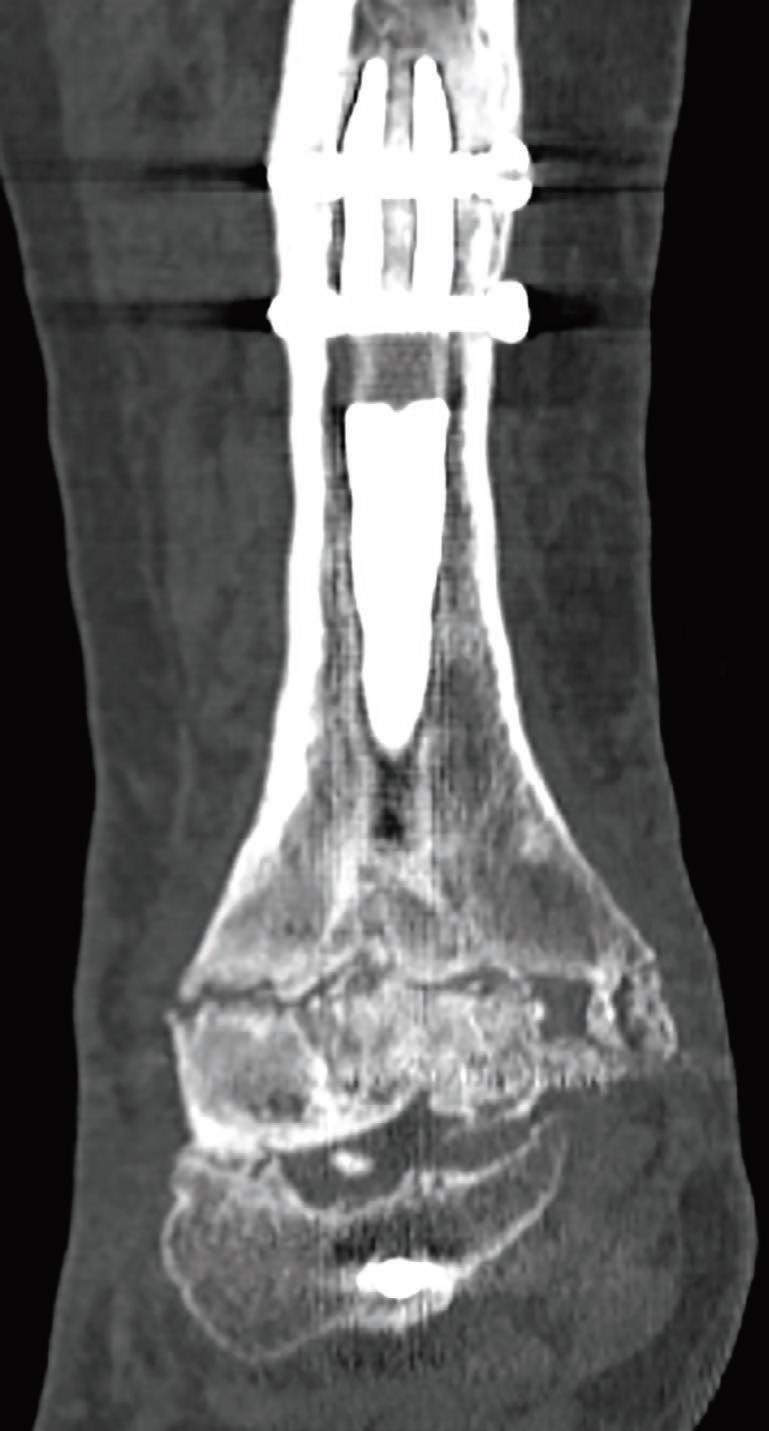
(c)

**Figure:**
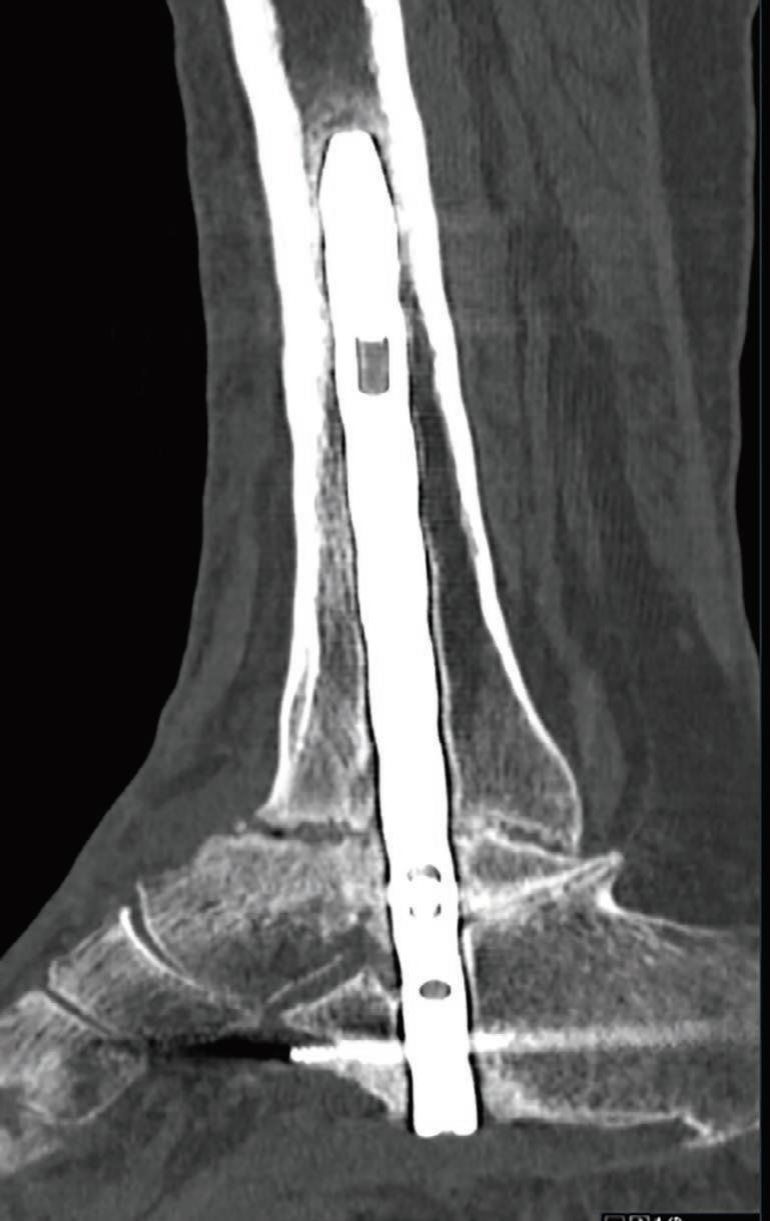
(d)

### 2.1. First Step: Bone Graft Extraction and Intramedullary Nail Removal

The procedure was performed in supine position, and cefazolin 2 g was administered as surgical antibiotic prophylaxis in accordance with the institutional protocol. A 5‐cm incision was made above the ipsilateral iliac crest, and a wedge‐shaped tricortical bone graft was extracted and stored in 0.9% saline solution.

The intramedullary nail and screws were removed using previous incisions and with intraoperative fluoroscopy. Absorbable sutures were used in subcutaneous to close the surgical incisions, and staples were used in the skin.

### 2.2. Second Step: Callus Debridement and Posterior Plate Implantation

The patient was then placed in a prone position with a support under the distal third of the leg and a lower thigh tourniquet. A longitudinal incision was made above the Achilles tendon, and the fascia and peritenon were opened and retracted. The tendon was split in line with its fibers, reaching approximately 6 cm from the myotendinous junction, and then, it was dislodged distally from its insertion, uncovering two tendon flaps that are separated with an autostatic retractor (Figure 2). The underlying structures are identified: Flexor hallucis longus (FHL) and posterior tibial tendon (PTT) were retracted medially, and peroneal tendons (PTs) were retracted laterally so that the pseudoarthrosis area was exposed.

Figure 2Intraoperative images. Trans‐Achilles approach (a). Achilles flap dissection (b). Underlying structures′ exposure (c). Plate placement (d). Split closure and bone attachment (e).

**Figure:**
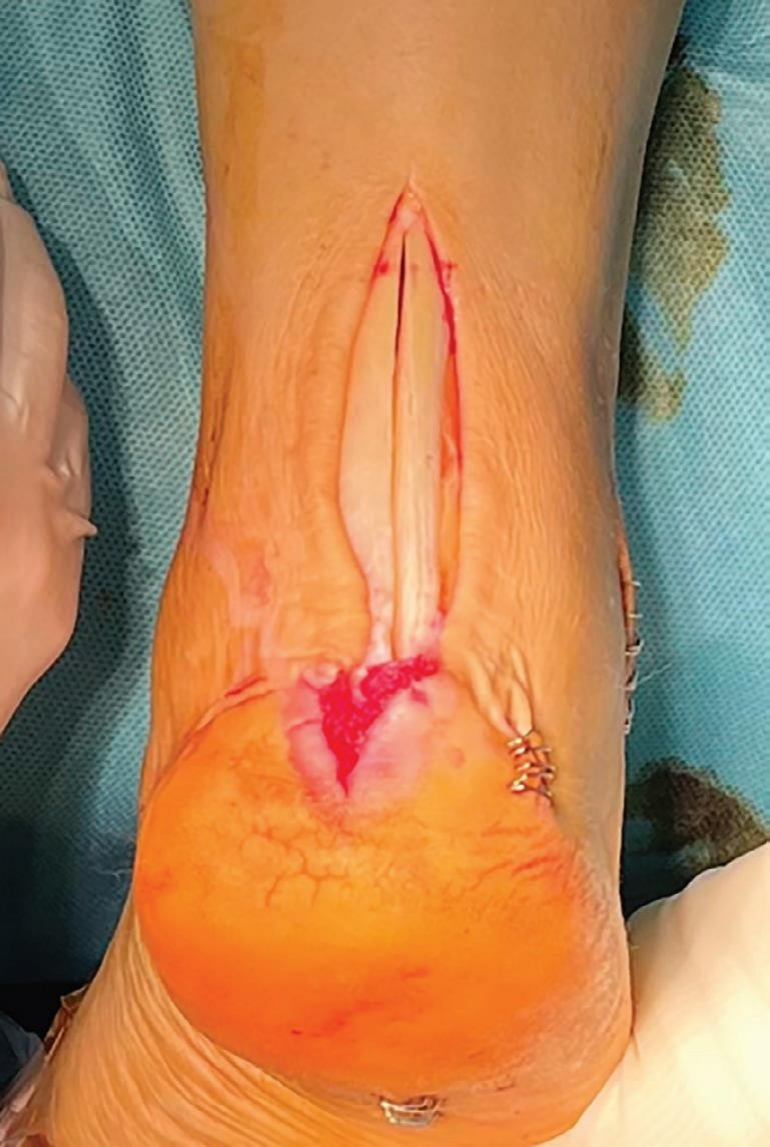
(a)

**Figure:**
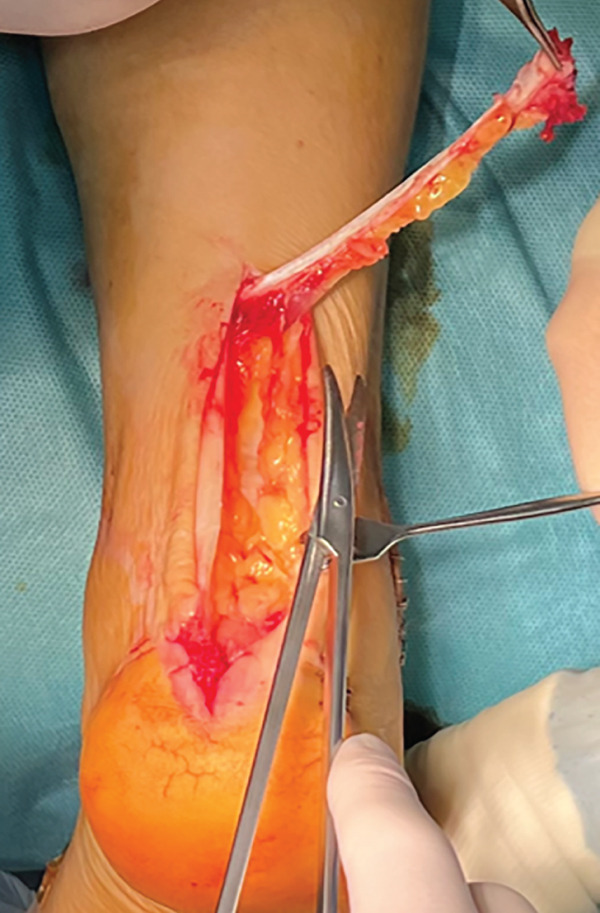
(b)

**Figure:**
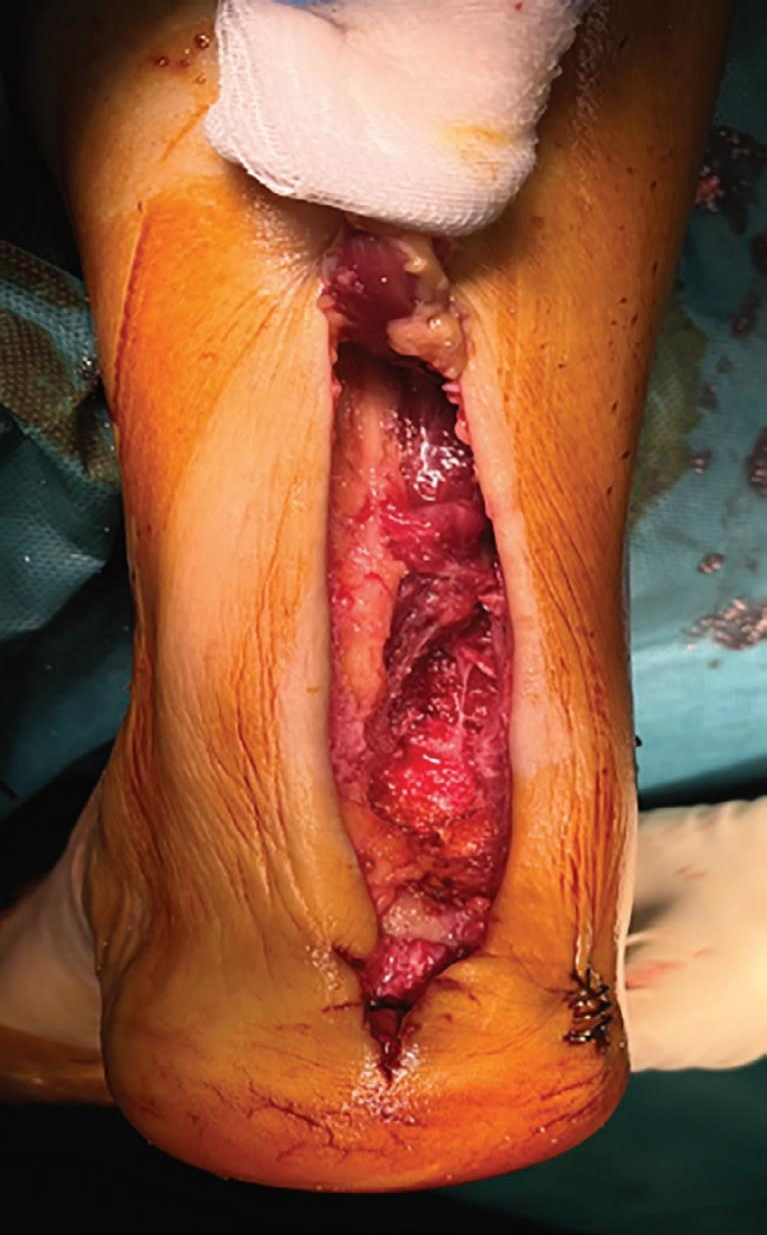
(c)

**Figure:**
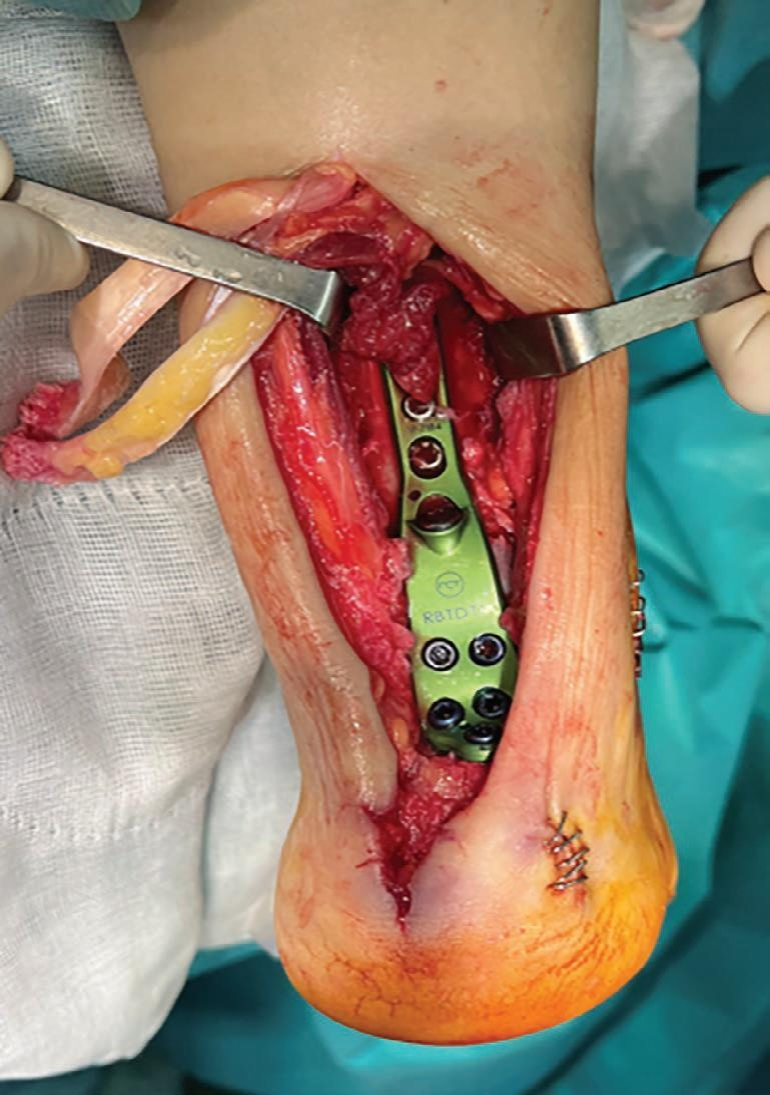
(d)

**Figure:**
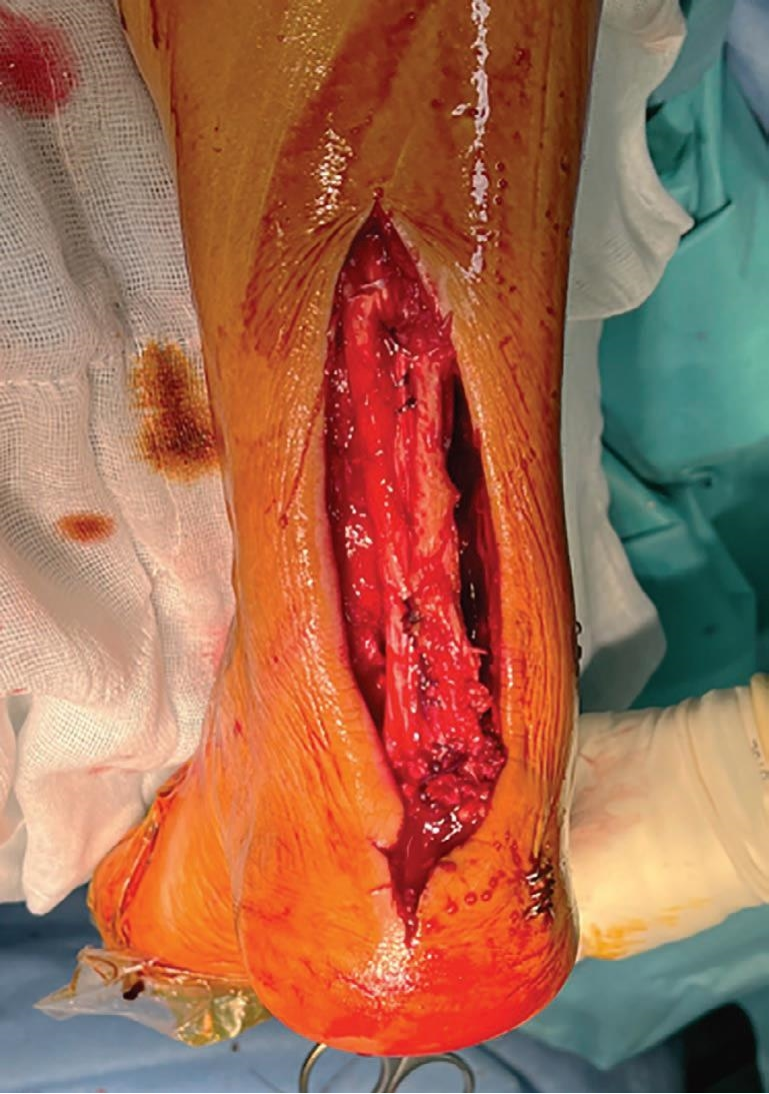
(e)

The pseudoarthrosis callus was debrided, and the remaining articular surface was removed using a conical milling cutter under intraoperative fluoroscopy. Intraoperative samples were obtained including both pseudoarthrosis site and the adjacent soft tissues and were submitted for microbiological analysis together with the osteosynthesis material. The bone then was milled and perforated with 1.5‐mm K‐wires until reaching bleeding subchondral bone. During the surgery, it was observed that subtalar arthrodesis had been achieved, so it was decided not to touch it. Bone defect was then filled with the autologous iliac crest bone graft collected before, mixed with cancellous allograft bone and I‐Factor (Cerapedics).

Subsequently, the ankle was placed in the desired position for the fusion (10° of external rotation, neutral dorsiflexion and 5° of valgus), and it was stabilized with an anatomical Activ Fuse standard plate (Newclip). First, we introduced a distal locking screw to the calcaneus body, followed by a proximal cortical one to the tibial metaphysis and the transfixing screw through the subtalar joint straight to the calcaneus. The osteosynthesis was completed by inserting three more proximal locking screws and four more distal ones, two in the talus and two in the calcaneus.

After a proper exam with fluoroscopy, the two Achilles tendon flaps previously split are attached, and the distal part is anchored to the bone with transosseous sutures (PDS 3/0). Paratenon and fascia are closed with absorbable sutures (Vicryl 3/0) and the skin with simple monofilament ones (3/0). A short leg plaster splint was applied in the operating room, immobilizing the ankle in neutral dorsiflexion.

### 2.3. PO Care

Empirical antibiotic therapy with daptomycin 800 mg every 24 h and ceftriaxone 2000 mg every 24 h was administered until culture results were confirmed negative.

After 6 weeks of immobilization with a short leg cast, it was replaced by an articulated walking boot until the second month, starting protected partial weight bearing on Week 9 after the intervention; total weight bearing without the boot was permitted after the third month. From the sixth week onwards, a rehabilitation program with progressive passive and active range of motion exercises was introduced.

Progressive fusion was evidenced on the x‐ray after the 12th week, and it was completely healed by the seventh month. Weight‐bearing pain was highly relieved during the 7 PO months. But in the eighth month, the patient complained about occasional nonweight‐bearing pain in the anterior surface of the tibia. There was a periosteal reaction above the plate in the x‐ray, so a CT scan was done, showing a peri‐implant stress fracture (Figure 3).

Figure 3Postoperative imaging exams. First week PO x‐ray, lateral view (a). Third month PO x‐ray, lateral view (b). Seventh month PO x‐ray, lateral view (c). Ninth month PO CT, showing the described peri‐implant stress fracture (d).

**Figure:**
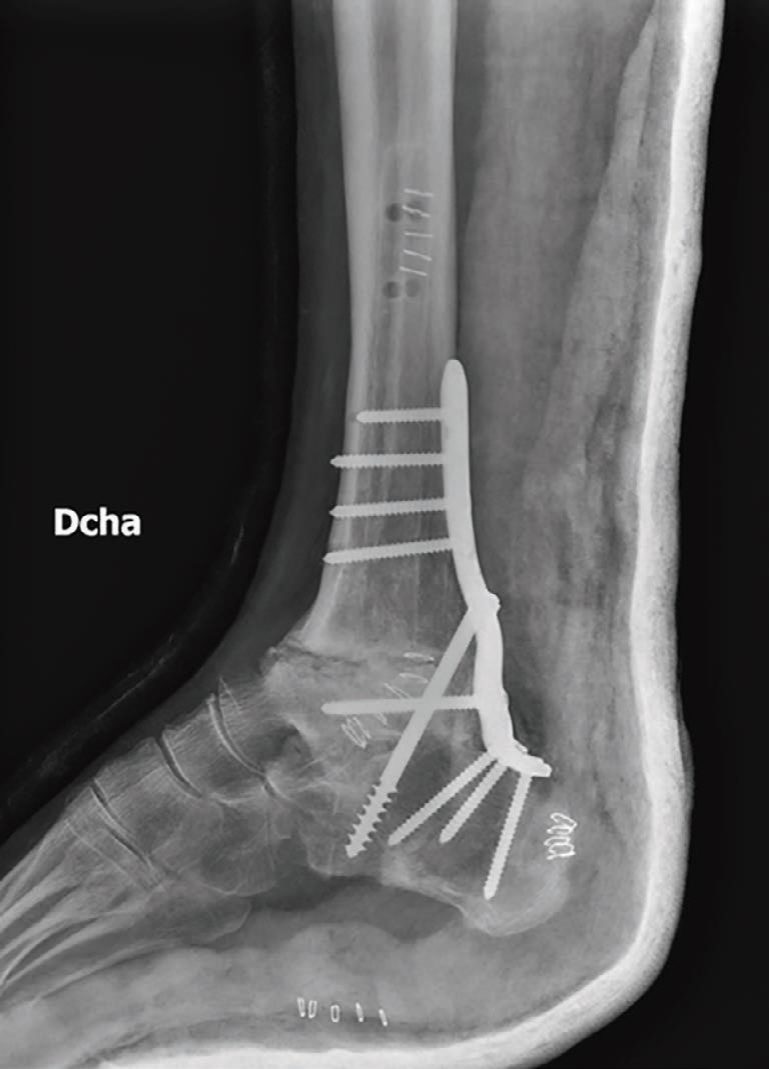
(a)

**Figure:**
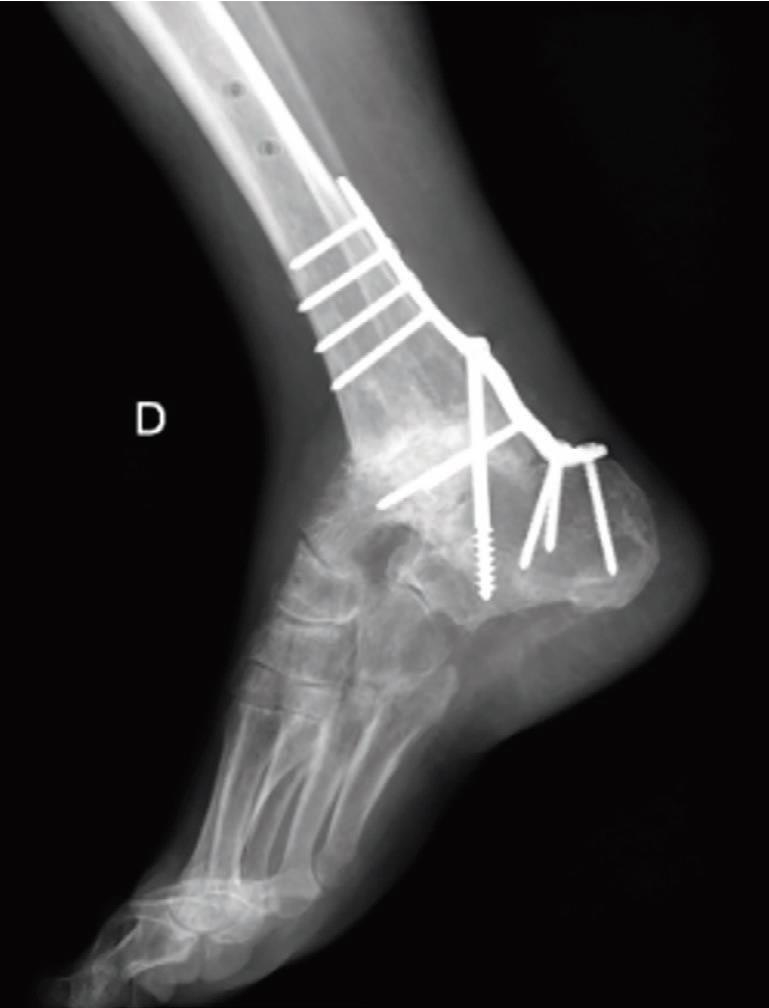
(b)

**Figure:**
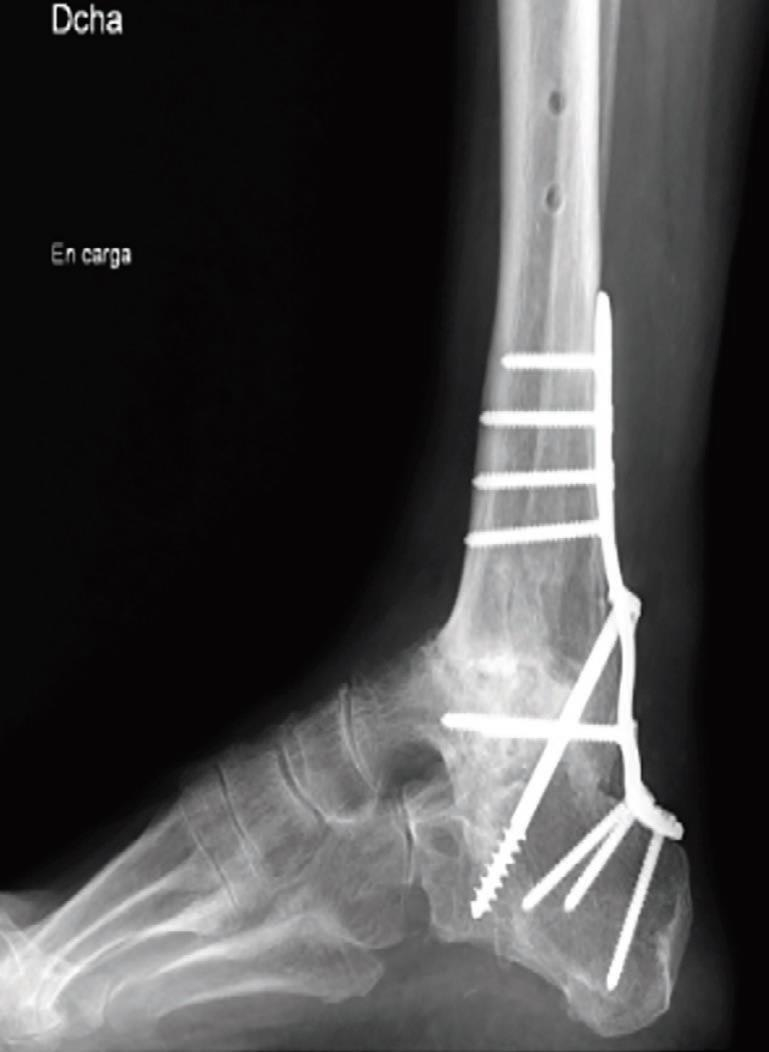
(c)

**Figure:**
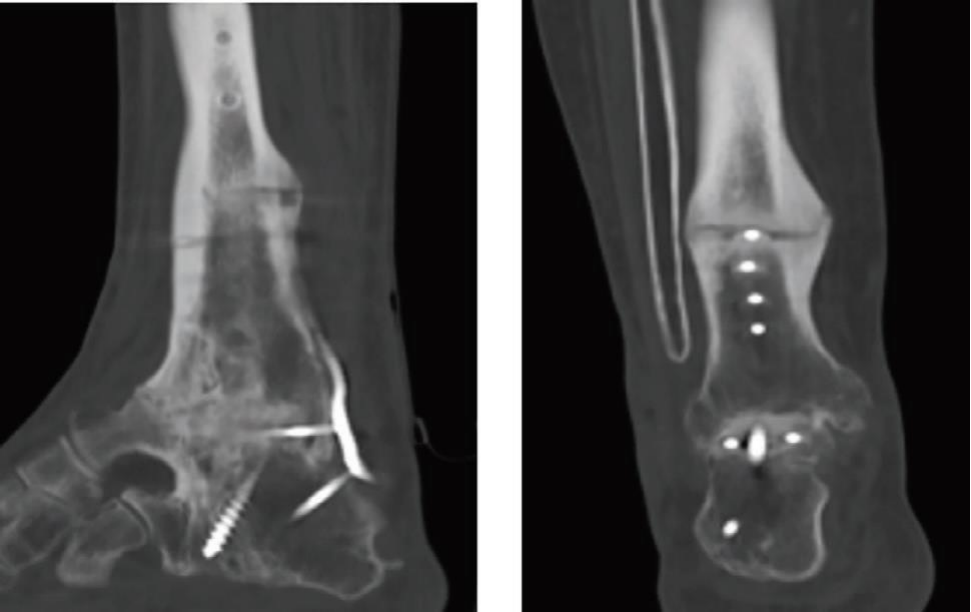
(d)

As the arthrodesis had been accomplished, it was decided to remove the implant.

A global PO follow‐up of 8 months was conducted, yielding good functional outcomes, with occasional pain and an AOFAS score of 77.

## 3. Case Presentation: Patient II

This case involves a 44‐year‐old male, with no relevant medical history, who underwent a tibiotalar arthrodesis with a nail (Expert Han, Synthes) in 2020, due to degenerative OA and who suffered from a PO necrosis of the talus and an arthrodesis nonunion (Figure 4). After 2 years, he was sent to our hospital where a tibiotalar arthrodesis was performed by a posterior approach to expose the talar necrosis area. We include a femoral condyle allograft too.

Figure 4Twenty‐two–month PO x‐ray after the first intervention, (a) anteroposterior and (b) lateral views. It shows the arthrodesis nonunion and the talar necrosis.

**Figure:**
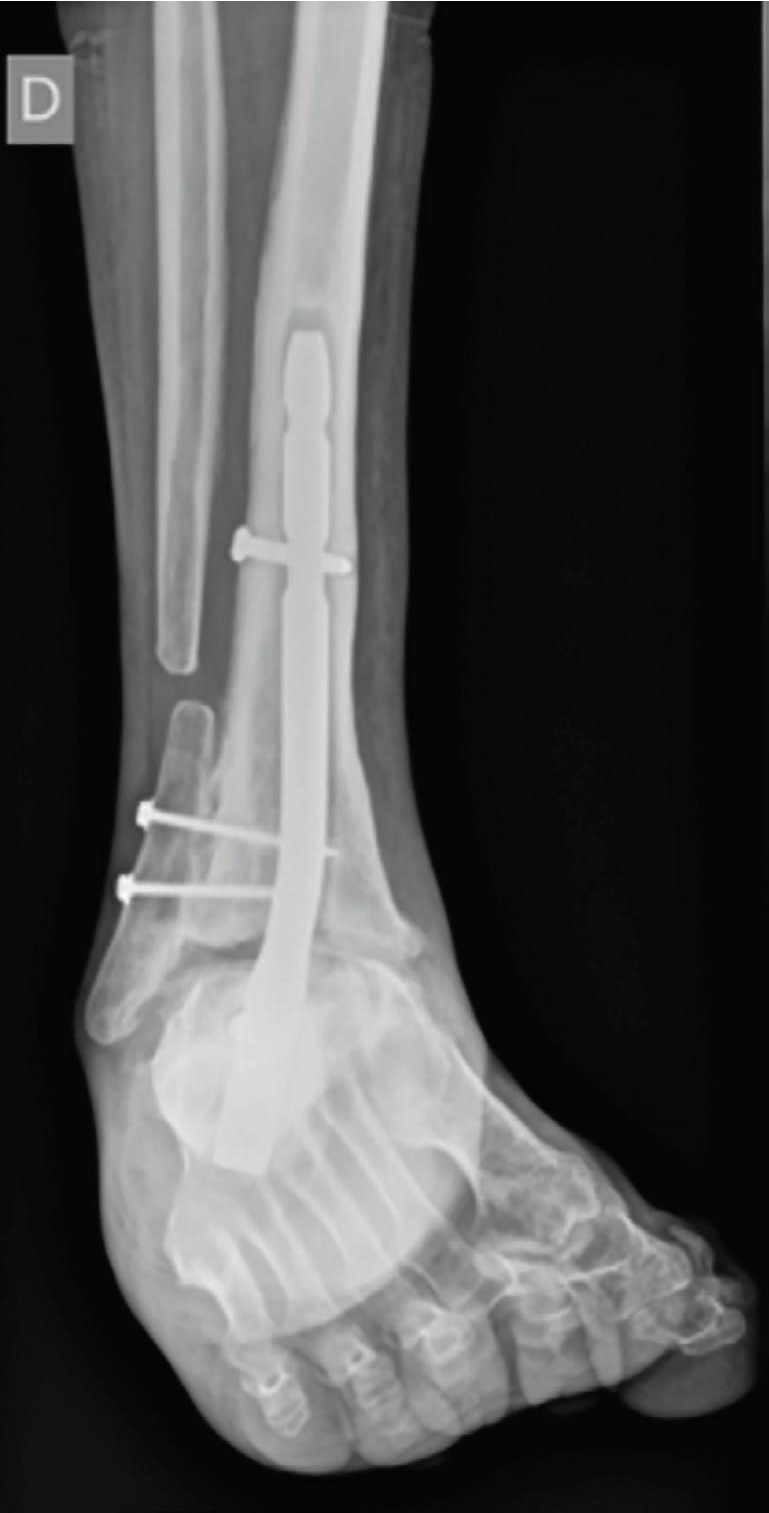
(a)

**Figure:**
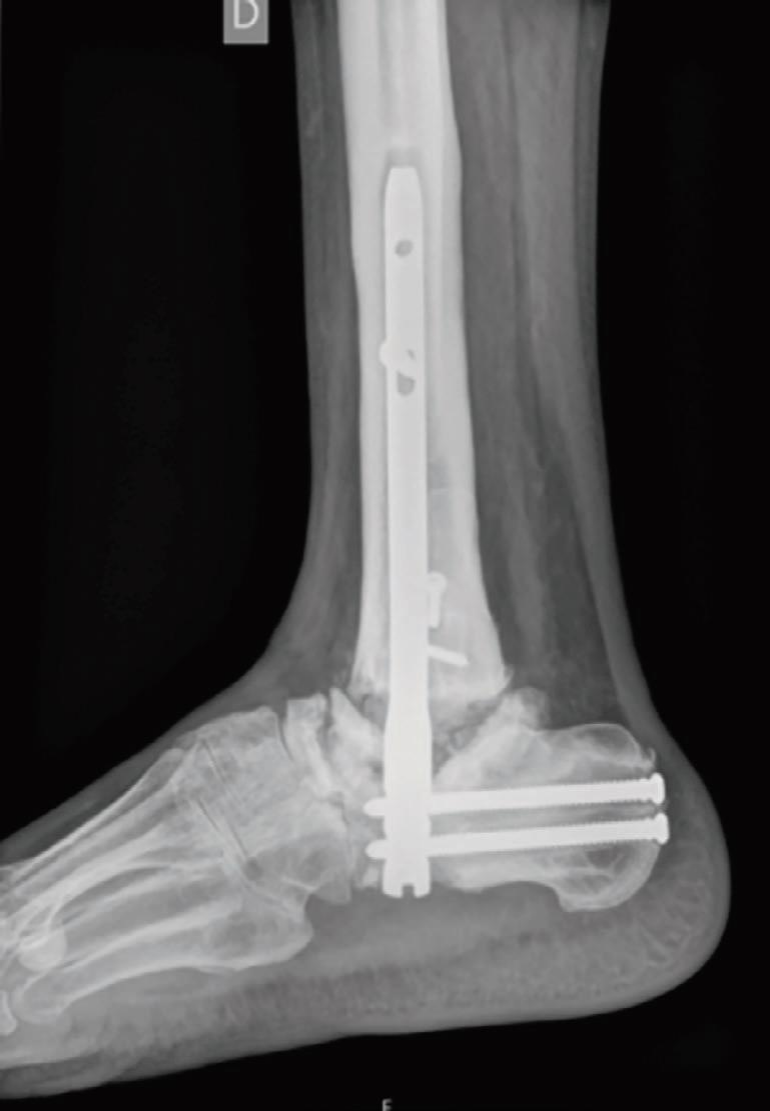
(b)

### 3.1. Surgical Technique

The intervention was carried out under general anesthesia in combination with a popliteal sciatic nerve block. First, the previous implant was removed, and then, the callus debridement and bone curettage were carried out. Lastly, an arthrodesis plate was implanted (Activ Fuse, Newclip).

The patient was placed in prone position, cefazolin 2 g was administered as surgical antibiotic prophylaxis, and a lower limb tourniquet was used. As the first step, a 3‐cm incision is performed over prior incision to remove arthrodesis nail before removing the screws. During the nail extraction, two screws were accidentally broken, and they were left in the tibial metaphysis.

Afterwards, a posterior longitudinal incision was made above the Achilles tendon. It was split and distally dislodged so that the pseudoarthrosis callus was exposed and debrided. A conical shaver was used to curettage the articular surfaces including the subtalar, and multiple perforations with a 1.5‐mm K‐wires were carried out on the talus and tibial surface, adding cortical petalization of the talar surface as a surgical gesture. Once a bleeding subchondral bone was reached, the bone defect was filled with I‐Factor (Cerapedics) and a wedge from a condylar bone allograft, being able to correct the preoperative ankle valgus.

The ankle was then placed in the desired position (10° of external rotation, neutral dorsiflexion and 0° of valgus), and the osteosynthesis was made with an anatomical Activ Fuse standard plate (Newclip), with 3 cortical proximal screws in the tibia, 1 transfixing screw through the subtalar joint, and 5 locking distal screws. After checking the desired position and fixation, two more supportive cannulated tricortical screws were placed in the fibula.

As there was a residual forefoot supination deformity, a dorsal open wedge osteotomy (Cotton osteotomy) and a wedge graft were added.

The Achilles tendon split was attached, the distal part was anchored and the paratenon and fascia were closed with absorbable sutures. The skin was closed with simple 3/0 monofilament sutures interspersed with staples.

A short leg cast was applied in the operating room, immobilizing the ankle in neutral dorsiflexion.

### 3.2. PO Care

After 6 weeks of immobilization with a suropedic plaster splint, it was replaced by an articulated walking boot until the fifth month, starting protected partial weight bearing on Week 7 after the intervention; total weight bearing without boot was permitted after 21 weeks. From the sixth week onwards, a rehabilitation program with progressive passive and active range of motion exercises was introduced. No complications were reported during PO follow‐up (Figure 5). The patient was discharged from orthopedic follow‐up 1 year after surgery, with good functional results and an AOFAS score of 74.

Figure 5Postoperative imaging exams. First week PO x‐ray, lateral view (a). Six‐week PO x‐ray, anteroposterior and lateral view (b). Sixth month PO x‐ray, anteroposterior and lateral views (c).

**Figure:**
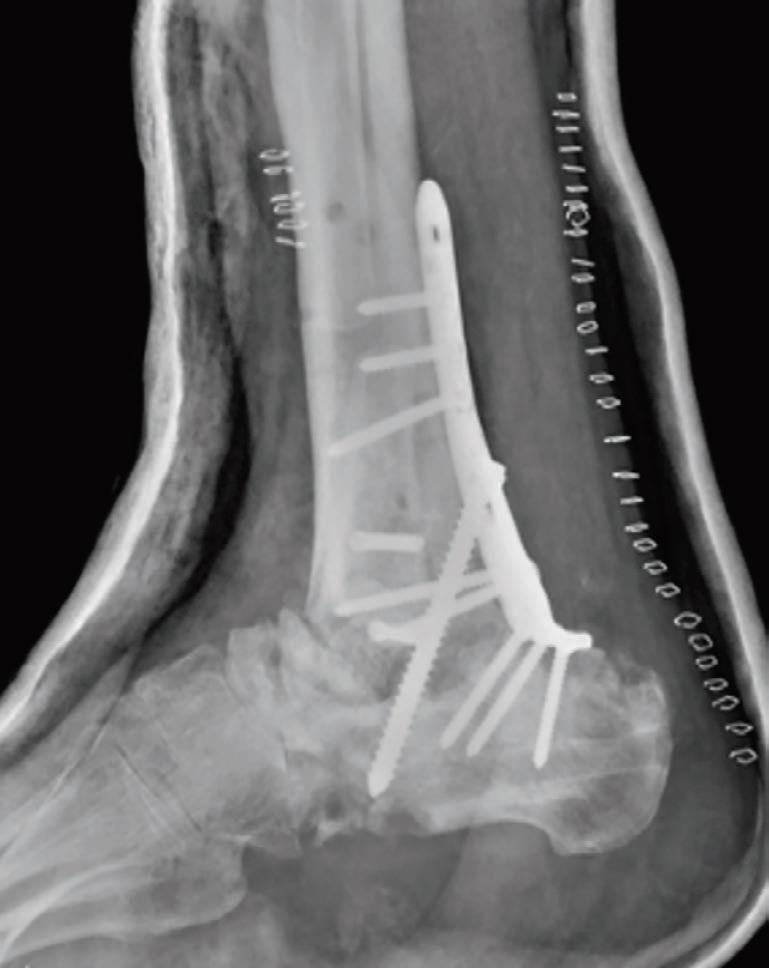
(a)

**Figure:**
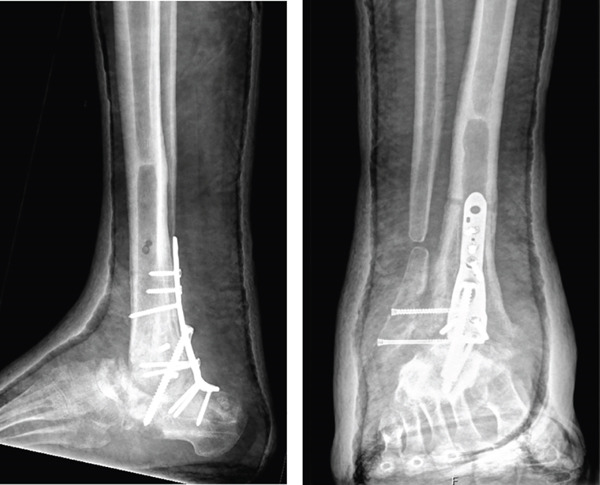
(b)

**Figure:**
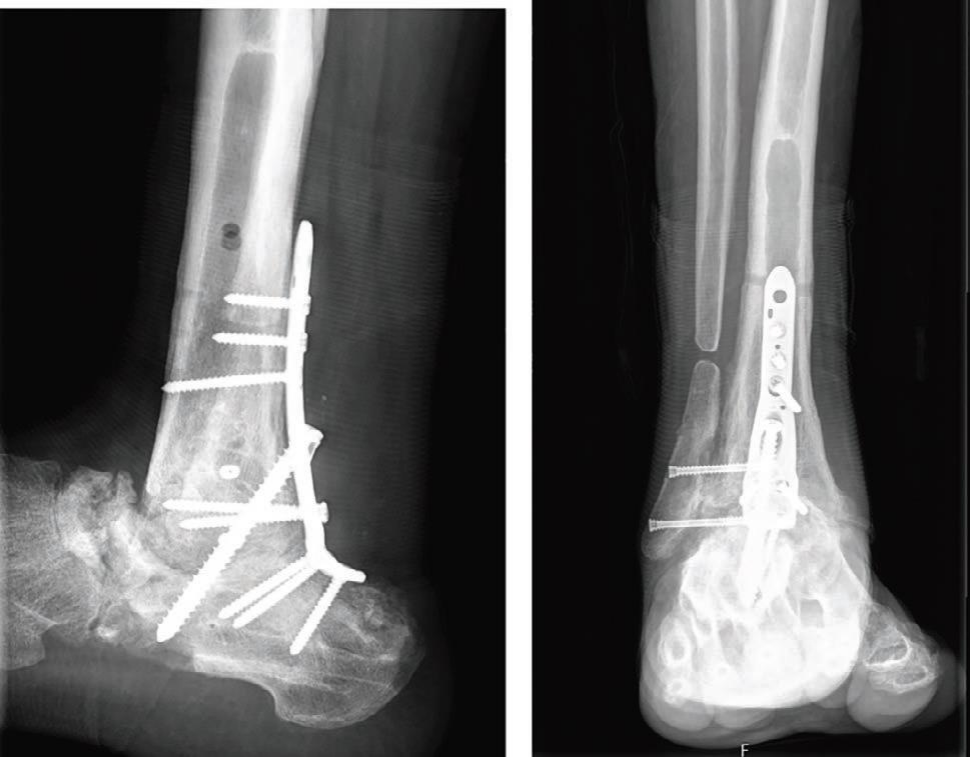
(c)

## 4. Discussion

The end‐stage ankle OA is a challenge for surgeons, even for the expert ones. TAR as well as tibiotalar and tibiotalocalcaneal arthrodesis are described in the literature as good treatment alternatives to solve this pathology. While TAR seems to be associated with a higher revision surgery rate, AA has a higher rate of complications in the short and middle term, increased discomfort related to implants, and a higher rate of peri‐implant fractures [10, 11]. Wats et al. compared both treatment options and described a revision surgery rate of 12.2% after 2 years in the group treated with TAR and 5.5% in the AA group and complication rates of 16% and 26%, respectively [12].

Thus, there are no clear indications of which patients are better for a TAR and which ones for an AA, although it is known that those suffering from obesity, diabetes, diabetic neuropathy, or physical high demands would have more benefits with a primary AA [13, 14].

The persistence of pain in relation to weight bearing after an AA should make us suspect that there is a complication. The union rate is described as 97.6% in patients with arthrodesis [15]. The pseudoarthrosis is quite common, and the revision surgery is a challenging operation with an important economic impact [16]. There are some risk factors related to a higher nonunion rate: diabetes, diabetic neuropathy, Charcot neuropathy, glycated hemoglobin > 7.5, and ASA > 2 [6, 17].

The AA after a failed TAR is also associated with higher nonunion rates, up to 13%, according to recent studies [18], and a revision rate of 25% in the first 3 years after surgery [19]. On the other hand, the revision surgery rate in patients with posttraumatic OA treated with AA is higher than in patients with primary OA. Ross et al. described a higher reintervention rate in relation to the need for implant removal and fusion of adjacent joints [20].

Previous arthrodesis of the adjacent joint is a risk factor to tibiotalar nonunion. Along these lines, the association of the subtalar joint fusion could be related to higher rates of complications and need for revision surgeries, according to the latest studies [21, 22]. On the other hand, neither the simultaneous arthrodesis of the distal tibiofibular joint nor the use of cancellous bone inserted in the arthrodesis has been shown to be related to an improvement in fusion rate [21], so they should not be associated in all cases. In patients suffering from avascular talus necrosis, the tibiotalocalcaneal arthrodesis is associated with delayed union rates of up to 32%, nonunion rates of up to 18%, and reintervention rates of up to 15% [23].

Regarding the risk of nonunion depending on the implant, there seem to be no differences in the rate of complications or in the rigidity of the osteosynthesis between using a plate or an intramedullary nail [24, 25], nor do there appear to be differences in the union rate depending on the approach used for the surgery [1].

On this topic, canulated screws appear to have fewer biomechanical benefits and a slightly higher rate of pseudoarthrosis when used isolated compared to when used in combination with other material [26, 27]. Gutteck et al. have described that osteosynthesis with a posterior plate has shown greater stiffness and stability in biomechanical studies compared to arthrodesis with lag screws [28]. Clifford et al. compared different arthrodesis techniques and concluded that the use of a lag screw in association with a plate (anterior or lateral) increases the stiffness contributing to the achievement of arthrodesis in comparison to the use of an isolated plate or isolated screws [27]. Zhang et al. suggested that a retrograde nail arthrodesis was associated with longer surgical procedures and greater blood loss, with earlier fusion rates compared to a locking plate laterally implanted, which could be associated with better fusion rates in patients with risk factors, although not significantly [29]. Rosemberg et al. have not found significant differences in the union rates performed with a retrograde nail compared to those performed with a lateral locking plate, nor in the rates of tibiotalar and subtalar pseudoarthrosis [24]. Reissing et al. have studied the fusion rates using a Phoenix nail, setting them at 79% for the tibiotalar joint, 70% for the subtalar joint, and 66% for both; and he has described a revision surgery rate of 10%, suggesting that wider nails could reduce the risk of implant failure and pseudoarthrosis [30].

With respect to the approach to be chosen, there are different options for the treatment of primary OA and its complications, and they are well described in the literature [31]. However, it is not so clear when talking about the revision surgery after a failed AA. Medial as well as posterolateral approaches allow good access to the joint, being the medial one the best to achieve the subtalar joint, and both are an interesting option to the classical lateral approach [19, 28]. The posterior approach is described as an alternative for the revision surgeries in patients who have had problems with soft tissues in the anterior, medial, and lateral parts permitting a complete coverage of the implants and reducing healing complications [32, 33]. Posterior trans‐Achilles approach allows good joint access in difficult cases and reduces the risk of healing problems [34]. Besides, the posterior plate seems to be useful in those patients with little healthy joint surface, for example, in the case of a talus necrosis [35]. In these patients, the posterior approach provides a better exposure of the distal tibia and talus compared to the classic posterolateral approach. Pellegrini et al. concluded that posterior trans‐Achilles arthrodesis has acceptable union rates (80.40%), regardless of the implant election (nail, plate, or screws), and it reduces the risk of vascular and cutaneous damage [36]. On the other hand, posterior locking plates have demonstrated good adaptation to the anterior and posterior surfaces of the bone [37, 38] and so they can be considered as a versatile option to the tibiotalar and tibiotalocalcaneal arthrodesis and its complications.

Different surgical alternatives are described to manage this pathology, including joint preservation approaches (percutaneous drilling, core decompression) and salvage procedures such as tibiotalocalcaneal arthrodesis with retrograde nail, tibiotalar arthrodesis with autologous tibial support (Blair technique), and arthroplasty. Arthroscopic arthrodesis with percutaneous screw fixation is reserved for patients with minimal talar dome collapse [39].

Although there are reports finding acceptable fusion rates in patients treated with retrograde nailing without biological support, bone graft association seems to improve union rates in the majority of the cases. Ramu et al. found union rates of 100% in patients with talar avascular necrosis treated by hindfoot arthrodesis and femoral head allografts [40]. On the other hand, Tenebaum et al. found similar fusion rates in a larger study in patients treated without structural allograft [41].

There is a growing interest about the reconstruction techniques for massive bone defect. In fact, 3D‐printed titanium implants seem to be an increasingly feasible alternative as a structural support technique after arthrodesis procedures in patients with avascular necrosis of the talus [42]. Dekker et al. described good functional results and satisfaction rates in 87% of the patients treated with customized 3D titanium implants for significant bone loss, including talar avascular necrosis and tibiotalar and tibiotalocalcaneal arthrodesis failures [43].

## 5. Conclusion

Surgery revision after a failed AA is a surgical challenge. Bone and soft tissue damage can be a significant limitation to plan the salvage procedure.

There are many investigations about different tibiotalar and tibiotalocalcaneal fusion techniques for primary OA and TAR failures, but scientific evidence about the treatment of an arthrodesis failure is less conclusive.

In the absence of a greater number of studies with a larger sample of patients and a longer follow‐up period over time, it seems that the trans‐Achilles rearthrodesis with a posterior plate after a fusion failure could be an appropriate option in selected patients, obtaining promising fusion rates with acceptable complication rates. Therefore, it could be a good alternative for patients with soft tissue damage where an anterior approach could jeopardize the osteosynthesis.

Even though talus avascular necrosis is a subject of growing interest, there is not much bibliography on treatment of nonunion after arthrodesis in these cases. A greater number of studies are needed on the application of posterior fixation both in primary surgery and rescue surgery after a failed arthrodesis.

## Funding

The authors received no funding for this work.

## Ethics Statement

The patients were informed and provided their consent to participate in this study.

## Conflicts of Interest

The authors declare no conflicts of interest.

## Data Availability

This article is based on the description of two clinical cases from our hospital and the review of the bibliography reflected on this paper. Data sharing is not applicable to this article as no new data were created or analyzed in this study.
